# The Effect of Low Survey Response Rates on Estimates of Alcohol Consumption in a General Population Survey

**DOI:** 10.1371/journal.pone.0035527

**Published:** 2012-04-20

**Authors:** Jessica Meiklejohn, Jennie Connor, Kypros Kypri

**Affiliations:** 1 Department of Preventive and Social Medicine, University of Otago, Dunedin, New Zealand; 2 Injury Prevention Research Unit, Department of Preventive and Social Medicine, University of Otago, Dunedin, New Zealand; 3 School of Medicine and Public Health, Centre for Clinical Epidemiology and Biostatistics, University of Newcastle, Newcastle, Australia; Brazil

## Abstract

**Background:**

Response rates for surveys of alcohol use are declining for all modes of administration (postal, telephone, face-to-face). Low response rates may result in estimates that are biased by selective non-response. We examined non-response bias in the NZ GENACIS survey, a postal survey of a random electoral roll sample, with a response rate of 49.5% (n = 1924). Our aim was to estimate the magnitude of non-response bias in estimating the prevalence of current drinking and heavy episodic (binge) drinking.

**Methods:**

We used the “continuum of resistance” model to guide the investigation. In this model the likelihood of response by sample members is related to the amount of effort required from the researchers to elicit a response. First, the demographic characteristics of respondents and non-respondents were compared. Second, respondents who returned their questionnaire before the first reminder (early), before the second reminder (intermediate) or after the second reminder (late) were compared by demographic characteristics, 12-month prevalence of drinking and prevalence of binge drinking.

**Results:**

Demographic characteristics and prevalence of binge drinking were significantly different between late respondents and early/intermediate respondents, with the demographics of early and intermediate respondents being similar to people who refused to participate while late respondents were similar to all other non-respondents. Assuming non-respondents who did not actively refuse to participate had the same drinking patterns as late respondents, the prevalence of binge drinking amongst current drinkers was underestimated. Adjusting the prevalence of binge drinkers amongst current drinkers using population weights showed that this method of adjustment still resulted in an underestimate of the prevalence.

**Conclusions:**

The findings suggest non-respondents who did not actively refuse to participate are likely to have similar or more extreme drinking behaviours than late respondents, and that surveys of health compromising behaviours such as alcohol use are likely to underestimate the prevalence of these behaviours.

## Introduction

Response rates for surveys of substance use in the general population show a steadily decreasing trend regardless of the mode of administration (postal, telephone, face-to-face) [1]. Studies with low response rates may produce prevalence estimates that are biased by selective non-response. That is, the chance that someone will participate in the survey may be related to the parameter being measured. A review of the alcohol survey literature has in some cases shown that non-respondents were heavier drinkers than respondents while other studies showed that non-respondents were more often abstainers than respondents, and in some cases, both heavy drinkers and abstainers were over-represented among non-respondents [2].

**Table 1:** Demographic distribution in non-respondents, respondents and total eligible sample.

Variable**	% Non-Respondents n = 1966*	% Respondents n = 1924*	% Eligible sample n = 3890*
	(95% confidence interval)	(95% confidence interval)	(95% confidenceinterval)
**Sex**			
**Men**	52.2 (50.0, 54.4)	44.1 (41.9, 46.4)	48.2 (46.6, 47.8)
**Women**	47.8 (45.6, 50.0)	55.9 (53.6, 58.1)	51.8 (50.2, 53.4)
**NZ Dep06**			
**1**	8.9 (7.7, 10.2)	13.2 (11.7, 14.8)	11.0 (10.0, 12.0)
**2**	9.1 (7.9, 10.5)	12.4 (11.0, 14.0)	10.7 (9.8, 11.8)
**3**	9.6 (8.3, 11.0)	11.8 (10.4, 13.4)	10.7 (9.8, 11.7)
**4**	9.0 (7.7, 10.3)	12.1 (10.6, 13.6)	10.5 (9.5, 11.5)
**5**	9.2 (8.0, 10.6)	11.0 (9.6, 12.5)	10.1 (9.2, 11.1)
**6**	9.8 (8.5, 11.2)	9.7 (8.4, 11.1)	9.8 (8.9, 10.8)
**7**	10.0 (8.7, 11.4)	8.9 (7.7, 10.3)	9.5 (8.6, 10.4)
**8**	10.9 (9.5, 12.3)	7.3 (6.2, 8.6)	9.1 (8.2, 10.0)
**9**	11.2 (9.8, 12.6)	8.0 (6.8, 9.4)	9.6 (8.7, 10.6)
**10**	12.4 (11.0, 14.0)	5.6 (4.6, 6.7)	9.0 (8.2, 10.0)
**Maori Descent**			
**Yes**	19.5 (17.8, 21.3)	10.4 (9.1, 11.8)	15.0 (13.9, 16.1)
**No**	80.5 (78.7, 82.2)	89.6 (88.2, 90.9)	85.0 (83.9, 86.1)
**Age**			
**18–24 Years**	14.6 (13.1, 16.2)	8.5 (7.3, 9.9)	11.6 (10.6, 12.6)
**25–34 Years**	24.6 (22.7, 26.6)	15.9 (14.2, 17.6)	20.3 (19.0, 21.6)
**35–44 Years**	22.7 (20.9, 24.7)	22.4 (20.6, 24.3)	22.6 (21.3, 23.9)
**45–54 Years**	19.5 (17.8, 21.4)	25.7 (23.7, 27.7)	22.6 (21.3, 23.9)
**55–64 Years**	13.6 (12.1, 15.2)	21.0 (19.1, 22.8)	17.2 (16.0, 18.4)
**65–70 Years**	4.9 (4.0, 6.0)	6.6 (5.5, 7.8)	5.8 (5.0, 6.5)

* Due to rounding percentages do not always add to 100% ** Where there was missing data for a demographic variable those individuals were excluded from that analysis

While the use of population weights to combat the impact of this non-response is widely used, this method simply weights prevalence estimates on the basis of the distributions of key variables in the respondents compared to those in the population/sampling frame. A more sophisticated model that considers the differences between groups of respondents as well as compared to the sampling frame may yield better prevalence estimates adjusted for non-response.

Lin and Schaeffer proposed the continuum of resistance model as an explanation for survey response behaviour. This model gives rise to two ways to estimate non-response bias in this study: to compare the demographics of respondents with non-respondents and establish whether they are different and secondly to establish whether “late respondents” are most like non-respondents [3].

The continuum of resistance model is only appropriate in cases where there is a strong relationship between demographic factors and the behaviour of interest. Given that alcohol use is strongly associated with demographic variables such as age, gender and socioeconomic status, comparing distributions of these characteristics between respondents and non-respondents gives an indication of the extent to which non-response may be selective [4]. This is only possible where the sampling frame contains information that can be used for comparison in the absence of a survey response.

The continuum of resistance model also proposes that the likelihood of response by sample members is related to the amount of effort expended by the researchers in order to elicit a response. This model suggests that those participants for whom the most time and effort is required to elicit a response (the “late respondents”) are more similar to non-respondents than are early respondents. Here we use the model to investigate non-response bias in the New Zealand arm of Gender, Alcohol and Culture: an international study (GENACIS).

**Table 2:** Comparison of demographic characteristics across response groups.

Variable**	% Early respondents (n = 1349)*	% Intermediate respondents (n = 362)*	% Late respondents (n = 204)*	% Total non-respondents (n = 1966)*	Subgroups of non-respondents	
					% Refusals (n = 457)*	*%RTS and unknown* *(n = 1509)* *
**Sex**						
**Men**	42.0	46.7	53.0	52.2	46.8	53.8
**Women**	58.0	53.3	47.0	47.8	53.2	46.2
**NZDep06**						
**1**	13.8	12.0	10.9	8.9	12.2	8.0
**2**	12.3	13.7	10.9	9.1	13.3	7.8
**3**	11.9	10.5	13.4	9.6	10.2	9.4
**4**	12.6	10.3	11.4	9.0	9.7	8.7
**5**	11.6	9.4	9.9	9.2	10.0	9.0
**6**	8.7	14.0	9.0	9.8	9.5	9.1
**7**	9.4	8.0	7.4	10.0	10.2	9.9
**8**	6.6	8.6	9.9	10.9	8.0	11.7
**9**	7.4	8.8	11.4	11.2	8.9	12.0
**10**	5.7	4.8	6.0	12.4	8.2	13.7
**Maori descent**						
**No**	90.7	89.2	83.3	80.5	88.2	78.2
**Yes**	9.3	10.8	16.7	19.5	11.8	21.8
**Age**						
**18–24 Years**	7.6	9.4	12.3	14.6	4.8	17.6
**24–34 Years**	14.3	16.0	25.0	24.6	12.7	28.2
**34–44 Years**	21.7	25.1	23.0	22.7	18.8	23.9
**45–54 Years**	26.1	27.1	20.1	19.5	23.6	18.3
**55–64 Years**	22.5	18.5	15.7	13.6	27.4	9.4
**65–70 Years**	7.8	3.9	3.9	4.9	12.7	2.6

* Due to rounding percentages do not always add to 100% ** Where there was missing data for a demographic variable those individuals were excluded from that analysis

## Methods

### Ethics statement

Ethical approval for data collection was given by the University of Otago Human Ethics Committee in January 2007.

### Design of the study

This was a cross-sectional survey of a nationally representative sample of New Zealand residents aged 18–70 years, who were on the combined (General plus Maori) electoral roll in 2007. The sample consisted of 4000 people randomly selected from the electoral roll. The alcohol and health questionnaire contained 100 items and took 20–30 minutes to complete. It covered the following areas: demographic information (age, sex and ethnicity), social networks, respondent's alcohol consumption, drinking contexts, drinking consequences, intimate relations and sexuality, violence and victimization, and health and lifestyle.

### Recruitment

The first contact with the participants was an introductory letter which outlined the aims and informed recipients that they would soon be sent a questionnaire. The letter also contained contact details (a toll-free number, email addresses and postal address) for the research team and asked recipients to make contact if they had any questions about the study, or if they did not want to participate.

The full questionnaire with a cover letter and an information sheet was sent two weeks later, with a request that recipients contact the research team if they did not wish to participate. As a small token of the research team's appreciation for recipients' consideration of the request to participate, a tea bag was sent out with each questionnaire. Previous trials have shown that the inclusion of token incentives increases participation [5].

About three weeks later, a reminder letter was sent to all sample members who had not yet responded, asking them to return their completed questionnaire, contact the research team for a replacement, or to decline to participate. If sample members failed to respond in four weeks they were sent a second questionnaire and letter.

Approximately six months after the initial contact, the study team obtained phone numbers for sample members who had still not completed the survey, by matching their name and electoral roll address with landline telephone listings. Where it was possible to find a telephone number, up to three attempts were made to contact the sample member and replacement questionnaires were sent out if necessary.

### Comparison of respondents with non-respondents and the target population

After ineligible participants were removed from the database, the remaining sample members were coded as either respondents or non-respondents. Age was estimated for members of both groups from the one year age bands given in the electoral roll. Indicators of sex, Maori descent and New Zealand Deprivation Index 2006 (NZDep06) deciles were also obtained for both groups from the electoral roll. The New Zealand Deprivation Index 2006 (NZDep06) was used an indicator of socioeconomic position. It is a small area deprivation measure, based on 9 items from the national census at the meshblock level. Meshblocks are the smallest unit of the census and include about 100 residents on average. NZDep06 deciles assign a score of 1–10 to particiants on the basis of their residential address, with 1 representing the least, and 10 the most, deprived 10% of the population. Distributions of these demographic characteristics were compared using chi squared tests. In assessing whether non-response was likely to have biased prevalence estimates, we also compared those who took part in the study with the target population.

**Table 3:** Estimates of drinking and binge drinking prevalence in the GENACIS survey.

Measure	Unadjusted prevalence estimates	Adjusted under the assumption of the continuum of resistance*	Adjusted using population weights
**Prevalence of drinkers (last 12 months)**	89.6%	88.7%	87.6%
**Prevalence of binge drinkers among current drinkers**	20.6%	25.5%	24.2%

* Assuming that RTS and unknown had the same prevalence as late respondents.

### Comparison of early and intermediate respondents with late respondents

The second analysis was a comparison of early, intermediate and late respondents. The determination of categories was non-arbitrary, relating to the effort required on the part of the researchers to elicit a response rather than the mere latency of response. Early respondents were those whose questionnaires arrived in the survey office before the first reminder was sent, intermediates were those whose questionnaires arrived before the posting of the second questionnaire, and late respondents were those whose questionnaires arrived after the second questionnaire was sent. These three groups were compared on current drinker status (yes/no) and binge status (yes/no).

Current drinkers were defined as having consumed alcohol at least once in the past 12 months. Those who were not current drinkers were classified as “abstainers” in this first analysis. We were particularly interested in drinkers who reported heavy episodes of drinking on a regular basis (binge drinking), namely, more than four drinks in a single drinking occasion, at least monthly. This cut off being a widely accepted measure of heavy episodic (binge) drinking [6].

The demographic characterisics of late respondents were also compared with all non-respondents, refusals, people whose survey was sent back marked return to sender (RTS) and people with whom the researchers had no contact (unknown), to test the assumption of the continuum of resistance model, i.e. that late respondents better resemble non-respondents than early/intermediate repondents. Sample members with missing information were excluded from this analysis. Data were missing in less than 5% of the total respondents.

### Adjustment of drinking measures

Prevalence estimates were recalculated for the population assuming that non-respondents who were RTS or unknown had the same drinking behaviour as late respondents. The prevalence of current drinkers and current drinkers identified as binge drinkers was also calculated using population weights (constructed using age, sex and NZDep06) to compare with the findings from the continuum of resistance model.

## Results

### Response rates

The overall response rate for this survey was 49.5% (1924 respondents/3890 eligible sample members). There were 1349 early (70%), 362 intermediate (19%) and 204 late (11%) respondents. Nine respondents could not be categorised by response time as we had no information on the date that their questionnaire was received.

### Comparison of respondents with non-respondents, and with the whole target population

Table 1 presents the demographic comparisons between the respondents and non-respondents. Statistically significant differences in the distribution of all demographic characteristics were shown between the two groups (p<0.001). Women were over-represented among respondents (55.9% vs. 47.8%), while Maori (10.4% vs. 19.5%) and 18–44 year-olds were under-represented (46.8% vs. 61.9%). Under-representation of younger adults was most marked in the 18–24 year age group. People from wealthier areas – indicated by a low NZDep06 score–were also over-represented among respondents: 48.6% of the sample had an NZDep06 score from 1–4 compared to only 36.2% of non-respondents.

Table 1 also shows the demographic comparisons of the respondents and the total eligible sample (respondents+non-respondents). Women were over-represented among respondents (55.9% vs. 51.8%), while Maori (10.4% vs. 15.0%) and 18–34 year olds (24.4% vs. 31.9%) were under-represented. People from poorer areas – indicated by a high NZDep06 score – were under-represented among respondents with 29.8% having an NZDep06 score of 7–10 compared with 37.2% of the total eligible sample.

### Comparison of early and intermediate respondents with late respondents

Table 2 summarises the comparison of the non-respondent groups with early, intermediate and late respondents. With the exception of NZDep06 decile it is apparent that late respondents most resemble the total non-respondent group and the group of non-respondents identified as RTS and unknown. By contrast, the non-respondents identified as refusals were similar to the early and intermediate respondents.

Among current drinkers it was found that late respondents were more likely to be binge drinkers than were early and intermediate respondents (31.5% compared to 18.7% and 21.9% in early and intermediate respondents respectively p = 0.001). The continuum of resistance model proposes that late respondents will better resemble non-respondents than early/intermediate respondents. As shown in Table 2, the late respondents are, as expected according to the model, most like non-respondents in their demographic characteristics but only for non-respondents who did not contact the investigators to decline. Given that alcohol is strongly associated with these variables, we can reasonably assume that RTS and unknown non-respondents would have similar drinking behaviour to late respondents, or perhaps more extreme. Accordingly, our results are likely to have underestimated the true proportion of binge drinkers in the population.

### Adjustment of drinking measures

We adjusted the drinking estimates using the assumption that the RTS and unknown respondents' drinking patterns were there same as late respondents'. Under this assumption, the prevalence of current drinking in the respondents was slightly lower (88.7% vs. 89.6%). Among current drinkers the prevalence of binge drinking increased from 20.6% to 25.5% when adjusted in this way.

Table 3 shows three measures of prevalence for current drinkers and current binge drinkers: the unadjusted prevalence, the prevalence adjusted under the assumption of the continuum of resistance model, and the prevalence adjusted using population weights. The estimates adjusted using the continuum of resistance are higher for both drinking and binge drinking compared to those adjusted using population weights. Notably, assuming that the continuum of resistance model is a better basis for adjustment, then population weights do not sufficiently adjust for non-response bias.

## Discussion

As expected, there were differences in the distribution of demographic characteristics between respondents and non-respondents. The respondents sample under-represented men, young people, people of Maori ethnicity and people of lower socioeconomic status. Comparisons of early, intermediate and late respondents showed that the three response groups differed significantly in their demographic characteristics and some drinking behaviours, with late respondents most likely to be binge drinkers. Both methods of adjustment suggested that the data underestimated the prevalence of binge drinkers, and that using population weights to adjust for non-response also underestimated the prevalence of binge drinkers compared to the conservative adjustment made under the assumptions of the continuum of resistance model.

The findings relied on self-reported alcohol consumption, which may be subject to information bias arising from recall error, miscomprehension, and deliberate misreporting. Notably, the demographic characteristics of the whole non-respondent group were similar to those of the late respondents but when divided by the type of non-response, people who took the time to decline the study invitation were more like early and intermediate respondents and it was the people with whom we had no contact (or had their invitation sent back marked return to sender) that most closely resembled the late respondents. Refusals were more likely to be female, older, from less deprived areas and of European ethnicity while the RTS and unknown group were younger men, from more deprived areas and were more likely to be of Maori descent. The adjustments of drinking prevalence using the continuum of resistance model are conservative, given that the unknown and RTS group may have even more extreme drinking behaviours than late respondents. However, this analysis did not make any adjustments for the refusal group, given their similarity to early and intermediate respondents.

Our findings are consistent with several previous studies of non-response. A web-based health behaviour survey of New Zealand tertiary students showed that those participants who responded latest reported more risk behaviours than early respondents, with a significant increase in the prevalence of binge drinkers in late respondents [7]. The results are also consistent with the findings of a 2002 study that examined non-response in an internet survey of alcohol use in students at a single university using the continuum of resistance model [2]. Finally, a study of non-response in a telephone survey of drinking also found that non-respondents were more likely to be male, young and heavy drinkers [8].

The findings from these studies suggest that even when response rates are not especially low, selective non-response may substantially bias estimates of drinking behaviour. As shown in the adjusted estimates in this study, the current practice of weighting prevalence estimates using population weights to adjust for non-response is likely to underestimate the prevalence of binge drinking in population surveys. This is predictable since it is the least well represented subgroups in the population that are the most altered by population weighting.

The findings from the demographic comparisons are consistent with the theory behind the continuum of resistance model, showing that late respondents have very similar demographics to non-respondents, although this appears to only be true for non-respondents who did not actively decline to participate. Adjusting for non-response conservatively by assuming the RTS and unknown non-respondents are the same as late respondents showed that this survey, and likely other population surveys of alcohol use with similar response rates, significantly underestimated the prevalence of binge drinking. Adjustment using population weights also showed that the study underestimated this prevalence, however, to a smaller extent than the estimate made using the continuum of resistance model. While the continuum of resistance model appears to give a better estimate of binge drinker prevalence than population weighting, this too could still be an underestimate if the non-respondents are even more extreme in their drinking behaviours than late respondents.

The continuum of resistance model may be a better option for adjusting for non-response in surveys of alcohol use, but we can only use this model for behaviours that are highly correlated with demographic information available for non-respondents, i.e. contained in the sampling frame.

The impact of non-response on study findings will be influenced by type of health condition being surveyed and therefore the motivations to respond to the survey. For example, studies of non-response in surveys of respiratory health found that people who were suffering from poor respiratory health were likely to be early respondents, while people who were current smokers were more likely to be late respondents [9], [10]. This suggests that when a behaviour under investigation is one that compromises health or is socially undesirable people with the risk behaviour are less likely to respond to the survey and therefore prevalence estimates will be underestimated if there is a low response rate.

For health compromising behaviour, such as binge drinking, further research is needed into the motivations for response and ways that participation from this group could be increased.

For surveys of alcohol use, the prevalence of harmful drinking patterns should be assumed to be underestimated when response rates are low. The findings of this study also suggest that the continuum of resistance model may be a more effective method of adjusting for non-response than population weights and that when examining the effect of non-response it may be useful to separate refusals from other non-responders as they appear to be a significantly different group. There is urgent need for methods to increase survey participation, especially in surveys of health compromising behaviours.

## References

[1] Caetano R (2001). Non-response in alcohol and drug surveys: a research topic in need of further attention.. Addiction.

[2] Kypri K, Stephenson S, Langley J (2004). Assessment of Nonresponse Bias in an Internet Survey of Alcohol Use.. Alcoholism: Clinical and Environmental Research.

[3] Lin I-F, Schaeffer NC (1995). Using survey participants to estimate the impact of nonparticipation.. Public Opinion Quarterly.

[4] Wechsler H, Dowdall GW, Davenport A, Castillo S (1995). Correlates of college student binge drinking.. American Journal of Public Health.

[5] Dillman DA (2000). Mail and Internet Surveys – *The tailored design method*..

[6] Wechsler H, Austin SB (1998). Binge drinking: The Five/Four Measure.. Jounal of Studies on Alcohol.

[7] Kypri K, Samaranayaka A, Connor J, Langley J, Maclennan B (2011). Non-response bias in a web-based health survey of New Zealand tertiary students..

[8] Wild TC, Cunnigham J, Adlaf E (2001). Nonresponse in a follow-up to a representative telephone survey of adult drinkers.. Journal of studies on alcohol.

[9] Hardie JA, Bakke PS, Morkve O (2003). Non-response bias in a postal questionnaire survey on respiratory health in the old and very old.. Scand J Public Health.

[10] Verlato G, Melotti R, Olivieri M, Corsico A, Bugianai M (2010). Asthmatics and ex-smokers respond early, heavy smokers respond late to mailed surveys in Italy.. Respiratory Medicine.

